# The relation of neuroticism to physiological and behavioral stress responses induced by auditory startle

**DOI:** 10.1002/brb3.2554

**Published:** 2022-04-11

**Authors:** Malcolm Sehlström, Jessica K. Ljungberg, Anna‐Sara Claeson, Markus B. T. Nyström

**Affiliations:** ^1^ Department of Psychology Umeå University Umeå Sweden; ^2^ Engineering Psychology Department of Health, Learning and Technology Luleå University of Technology Luleå Sweden

**Keywords:** cognition, neuroticism, reaction time, startle, stress

## Abstract

**Introduction:**

The negative cognitive effects of the startle response are not yet fully understood. Ecological observations in the aviation field indicate risk for severe outcomes in complex or pressured situations, while sparse previous research suggests milder negative effects on simple cognitive tasks. Neuroticism is proposed as a factor related to the level of negative effects following startle.

**Methods:**

This study examined the effects of startle on performance in a choice reaction time task and analyzed relations between performance, neuroticism, and physiological stress.

**Results:**

Our results indicate that reaction time directly following startle was not affected, but reaction time in subsequent trials was significantly slower. Neuroticism and physiological stress were both unrelated to this performance effect.

**Discussion:**

We argue that higher complexity/demand tasks are necessary to complement the research on base cognitive functioning in relation to startle. If neuroticism is related to startle effects, this is more likely to be found in these higher demand situations.

## Graphical abstract

This study examined the effects of startling sounds on sustained attention and the relation of this to neuroticism and physiological stress responses. Attention performance was affected slightly, but this effect was unrelated to trait neuroticism and stress.

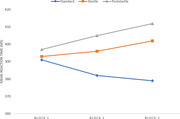

## 1 INTRODUCTION

The startle response is a defense mechanism which is triggered in response to sudden, intense stimuli, such as unexpected and loud sounds or movements (Koch, 1999). It exists in humans and other animals and is a quick reflexive process to protect the organism (Davis, 1984). The response includes the characteristic flinching reflexes and increased heart rate and blood pressure. The stimuli is also subjected to cortical threat assessment (Davis, 1992; LeDoux, 2000), from which additional subsequent physiological effects may follow. In a survival sense, it is better to react too often than not at all, and this mechanism to respond and simultaneously assess the need for further defensive action has become easily triggered, regardless of whether there is an actual threat or not. Studies (Davis, 1984; Yeomans & Frankland, 1995) of the involved reflexive actions, such as the blink reflex, have revealed phenomena like prepulse inhibition (Graham, 1975) and involuntary triggering of prepared motor actions (Valls‐Solé et al., 2008). There are, however, still gaps in the knowledge of cognitive effects following startling stimuli. The way that the startle response affects basic and higher cognition could be informative for the understanding of effects following startle in threatening real‐life scenarios, such as within the field of aviation. This field is experiencing increased interest and focuses on the effects of startling events on pilots’ performance. For instance, a review by Martin et al. (2015) noted incorrect decision making following a startling event was the probable cause of a number of aircraft accidents in recent years. Efforts are being made to improve the understanding of this phenomenon and to improve safety where there are human operators in safety‐critical situations.

The startle response is strongly connected to the fight or flight response. When a startle response is initiated, signals are sent to the brain to conduct a threat assessment (Davis, 1992; LeDoux, 2000). It is assumed that part of the cognitive effects following a startling event arises from the large stress response triggered by the fight or flight response, which tells humans that they are in serious or mortal danger. In much laboratory research, ecological validity like this can become an issue. As the threat‐potentiation aspect of the startle response seems to lead to long‐lasting stress and greater cognitive effects (Dismukes et al., 2015; Martin et al., 2015), it becomes very hard to concretely examine the effects of this scientifically. It is not ethically acceptable to bring participants into a lab and induce a startle that is comparable to one experienced in actual threatening scenarios.

However, physiological and cognitive effects are known to accompany startle responses without the presence of any threat. This implies that simply the presence of the startle reflex and the subconscious signal processing of the threat assessment appear to be enough to negatively affect cognitive performance on some level in some visual tasks (Thackray, 1988). Thackray (1988) noted that participants in a visual tracking task displayed impaired psychomotor performance that lasted for 5 s after being exposed to a startling acoustic stimulus. In a startle study by Vlasak (1969), the key aim was to examine the effects of startling sounds on performance in a visual tracking task characterized by even fewer cognitive demands than the one in a study by Thackray (1965). Vlasak (1969) observed that performance was only impaired for 2 s following a startle. In the same study, the author also reported that an increase in the demands for mental task had a greater negative effect on performance. Similar effects were found in a study by Woodhead (1959) in which negative effects on geometric sorting task performance lasted as long as 30 s after the onset of the startle stimulus.

While the previously mentioned research has shown negative, slowing effects on cognitive performance following a startle response, a circumstance in which a startle can improve reaction time has also been discovered. Dubbed the StartReact effect (Valls‐Solé et al., 1995), it has been shown that a prepared motor action (such as pressing a button or bending one's wrist) can be initiated involuntarily by a startling sound and thus performed with improved speed. This phenomenon is not completely understood, but it is argued by some to happen because of the subcortical startle reflex pathways activating the motor system (Smith et al., 2019). The presumption for a StartReact effect is the setting of simple reaction time, which implies a response is to be carried out in response to a single trigger without any further analysis or decision making. While the underlying mechanisms of the StartReact effect are still being studied, the negative effects of startle on reaction time when startle is used as outside of a simple reaction paradigm are less examined. Indeed, what are the effects of startling stimuli on reaction time when used as distractors, rather than as impetuses to trigger a StartReact effect?

In the few existing studies, it has been argued that these examinations of startle in nonthreatening scenarios can still grant valuable insight into its effects as both threatening and nonthreatening startle rely on the same mechanisms, although the effects produced will be of a lesser magnitude (Thackray, 1988). Even so, few studies other than those mentioned have been conducted on the cognitive effects of startle. Thus, with this study, we aimed to continue this line of research by picking up and examining the effects of startle on basic cognition. Regarding the interaction between the startle response and cognitive functioning, there is very limited theorizing at this time. In the related field of distraction, theories such as the load theory of attention (Lavie et al., 2004) explain performance variation through the demands placed on perception and cognition. Interruption research suggests that after a task is interrupted, which startling sounds must be argued to do, recovery time is needed for the cognitive system to recover the mental context to perform the task, depending on complexity (Altman & Trafton, 2007). This can be related to the interruption hypothesis of startle, as posed by Blumenthal et al. (2015). This hypothesis suggests that the startle response itself may interrupt information processing, although the workings of this is yet to be understood. We assume a partial overlap from distraction and interruption research to the effects of startle in this study, but these concepts cannot to be equated, however. The initial stimuli might be distracting or interrupting, but granted the processes of the startle response; it is likely that the processing and physiological response is burdening the system. Although the attention‐capturing distractive nature of a startling sound can be expected to account for some of possible performance effects, the reflex, threat assessment, and stress responses do not follow from distraction.

Sternbach (1960) and Thackray (1965) noted that some participants were significantly slowed in their response to a startling stimulus, and some responded relatively quickly; there was some level of individual variation in how it affected participants. The authors argued a partial explanation for the variation was the indication that a higher level of physiological response to the stimuli was connected to a greater slowing of the response. Individual variation in response to a startling stimulus, although not well examined in regard to cognitive performance, has been well examined when it comes to the startle reflex part. The variation in startle reflex magnitude is a well‐researched aspect, and it has been shown that the startle reflex magnitude can vary with emotional state, neuropathological states, and type of personality (Dawson et al., 1993; Lang et al., 1990; Wilson et al., 2000). The most studied and used conceptualization of personality today is the five‐factor model (McCrae & Costa, 1987). The model consists of five factors, or dimensions (i.e., openness, neuroticism, consciousness, agreeableness, and extraversion) that are used to understand an individual's personality structure (McCrae & Costa, 1987). The factor that has been most strongly related to how an individual copes with stressful and threatening events is neuroticism (Orleans‐Pobee, 2017). Neuroticism also relates to the tendency to experience negative affect (Larsen & Ketelaar, 1991) and to higher experienced stress levels (Schneider, 2004). It has been reported that individuals high in trait neuroticism tend to display larger startle reflexes (Wilson et al., 2000), show more conditioning effects in terms of affective learning, and tend to spend more attentional resources on fearful stimuli (Hur et al., 2016). Indeed, additional study results have suggested relations between anxiety (a subfactor of neuroticism), attention, and threat‐sensitivity (Sarapas et al., 2017). In the cognitive realm, neuroticism has been suggested to, and found to, come with increased susceptibility to distraction (Eysenck & Graydon, 1989; Szymura & Wodniecka, 2003). As neuroticism is connected to these factors and mediates the startle reflex, and a tentative connection between physiological response to startle and cognitive effects has been noted (Thackray, 1988), this study accounts for a possible startle effect interaction by explaining both neuroticism and the physiological stress response from startling stimuli. Well‐recognized physiological stress measurements such as the skin conductance response (SCR) and measures of heart rate variability (HRV) have indeed been used in the study of stress following startle (Grillon & Ameli, 2001; Ruiz‐Padial et al., 2003) and in the study of neuroticism (Di Simplicio et al., 2012; Norris et al., 2007). A higher degree of neuroticism is connected to a larger SCR and lower HRV in emotional processing, which could be expressed in a higher stress response following startle.

Finally, although the reflex part of the startle response is known to habituate over repeated exposures (Bradley et al., 1993), the extent to which habituation applies to cognitive effects is unclear. Previous studies have mentioned decreases in cognitive effects across exposures (Thackray, 1988), but these have not been properly examined as the studies relied on participants being exposed to only two startling stimuli, separated by some period of time. Thus, in this study, we employed a methodological design to include a larger number of startling stimuli, allowing us to increase the power of the data.

In sum, based on previous research, this study aimed to examine the interruptive effects of a startling acoustic stimulus by using a choice reaction time task to measure performance in sustained attention. We also wanted to examine individual variation in effects of startle in relation to personality and stress.

## 2 RESEARCH QUESTIONS

This paper addresses the following questions:

1. Do unpredictable startling acoustic stimuli affect performance on a simple choice reaction time (CRT) task and physiological stress responses?
  - If so, does this effect persist over time?
2. Is there a relation between startle‐affected performance, neuroticism, and stress responses?

## 3 METHOD

### 3.1 Participants

Thirty‐eight individuals (16 male and 22 female) participated. Participants were recruited through posters placed on the grounds of Umeå University and through lecture announcements that introduced the option to join the study. The mean age of participants was 27 (SD = 6.93). Exclusion criteria included hearing impairments, reported sensitivity to sounds, attention deficit disorders, and not having normal or corrected‐to‐normal vision. Displaying abnormal physiological stress data, or data implying lack of responsivity to startling sounds also warranted exclusion, but no participants had to be excluded on this basis. The study was accepted by the regional ethics board (Dnr: 2019‐02908). Participants were paid 200 SEK for their participation.

### 3.2 Material

#### 3.2.1 Physiological stress response measures

To measure the response to the startling stimuli, the Biopac MP150 with separate ECG and SCR modules was used. Physiological data were handled with the AcqKnowledge data software by BioPac. The ECGs were measured using a two‐lead setup with chest electrode placement. The ECG signal was treated with a band‐pass finite impulse response filter, with a low frequency sampling rate of 0.5 Hz and a high frequency sampling rate of 35 Hz. Heart rates were extracted with built‐in cycle functions, as were HRV. There are numerous specific time‐domain measures of HRV used in stress research (see Kim et al., 2018, for a review), and this study relied on a high frequency band HRV (HF‐HRV) measure of standard deviation between normal‐to‐normal intervals (SDNN), which was extracted in 5‐min intervals. SCR was measured by attaching electrodes to the fingers of the participant's nondominant hand. Signal transmission was facilitated with the use of an electrolyte electrode gel (Signa‐Gel 15‐60, electrode gel by Parker). The SCR data were treated with a low‐pass finite impulse response filter with a 1 Hz cutoff, followed by a high‐pass infinite impulse response filter with a 0.05 cutoff. Event‐related SCRs (ER‐SCR) for each stimulus were extracted using built‐in cycle functions.

#### 3.2.2 Personality assessment

Neuroticism was measured with the Mini International Personality Item Pool scales (Donnellan et al., 2006). The scales measured the constructs following the five‐factor model (openness, conscientiousness, extraversion, agreeableness, and neuroticism) and used a 20‐item short form variant of the 50‐item International Personality Item Pool (Goldberg, 1999). Each item was a statement where participants rated their agreement (e.g., “I am the life of the party” or “I sympathize with others’ feelings”). Ratings were made using a 5‐point Likert scale, ranging from 1 (*strongly disagree*) to 5 (*agreeing completely*). These short‐form scales have been proven to be strongly correlated with longer tests: For the neuroticism trait, Donnellan et al. (2006) reported the consistency analysis from a number of studies with Cronbach's alpha between .68 and .78.

#### 3.2.3 Digital environment

The reaction time paradigm was programmed in Matlab (2018) and run on a 64‐bit Windows 10 operating system. The computer screen used had a refresh rate of 60 Hz. Stimuli were presented through Vic Firth Sih1 headphones.

### 3.3 Design

**FIGURE 1:**
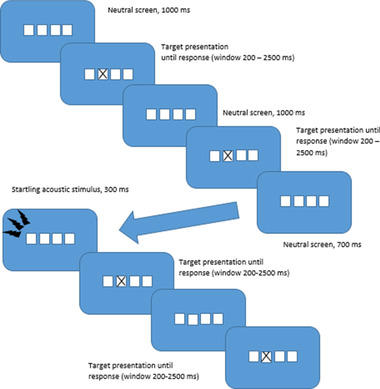
Choice reaction time task flowchart

The study followed a within‐subjects design in that all participants were exposed to the same testing procedure. The CRT task followed the general design of the Deary‐Liewald CRT task (Deary et al., 2011), with some modifications. In the Deary‐Liewald CRT task, four static squares are presented in the middle of the screen, and in each square, a target in the shape of a black cross might appear (see Figure 1). Using predetermined keyboard keys, participants were to respond as quickly and as accurately as possible by pressing the corresponding keys. Two measures of task performance, reaction time as well as accuracy, were recorded for use in analysis.

In order to be able to examine the possible effects of subsequent startling stimuli on reaction time, while allowing for enough recovery time between each stimulus, a design was decided upon where participants would be exposed to four startling stimuli over 300 task trials. This was considered one block and was repeated two times after brief rest periods of 2 min, for a grand total of 12 startle exposures and 900 reaction time trials over three blocks. This adapted in response to previously mentioned studies (Thackray, 1988), in which sometimes as few as two stimuli were used. Which trials were accompanied by stimuli was decided in a quasirandomized manner. True randomization was not utilized as to make sure that the stimuli were not presented too close in time to each other, and parameters were used to make sure that the stimuli were not presented with similar timing across the blocks. All participants were exposed to the stimuli in the same trials. The startle stimulus utilized was a previously tested 40 kHz 105 dB(A) white noise burst with instantaneous rise time, lasting for 300 ms and delivered binaurally through the headphones. White noise is the most commonly used startle signal, and short or instantaneous rise time are most effective and commonly used in startle research (Blumenthal et al., 2005). The stimulus was delivered 300 ms before an upcoming trial.

### 3.4 Procedure

**FIGURE 2:**
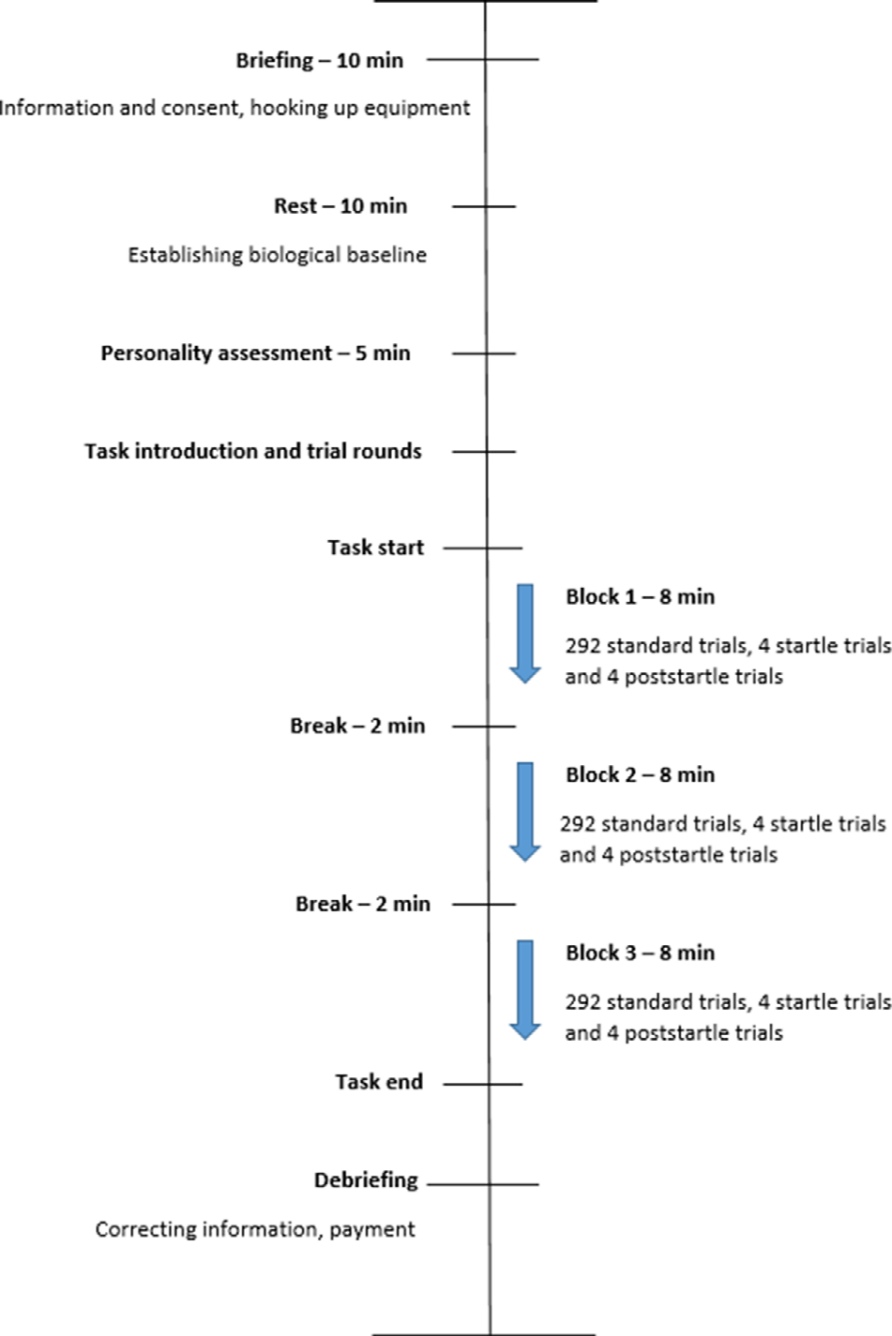
Visual representation of the data collection procedure

A visual overview of the procedure is provided in Figure 2. At the start of the study, participants were given written and oral information about the task and procedure. After receiving this information, participants signed a consent form. Next, the equipment for recording the ECG and SCR was attached, and a baseline was established, while the participants were at rest. Participants then completed the Mini International Personality Item Pool scales before moving on to the CRT task. Participants were seated in front of a computer screen at a distance of 60 cm and introduced to the task, completing a practice session of 40 trials with no stimuli presented. After finishing the practice session, participants were told that they could be exposed to sounds during the actual task, but that the sound was irrelevant to their task performance. After completing the three blocks of the task, participants were debriefed on the actual study design and purpose. The average participation time was 60 min.

### 3.5 Data analysis

An analysis was conducted using IBM SPSS Statistics (Version 26). For task performance, the trials were divided into three categories or trial types. Trials accompanying a stimulus were categorized as startle trials, those following stimuli as poststartle trials, and all others as standard trials. In order to examine reaction time and accuracy for the different trial types over time, 3^(Block: 1, 2, 3)^ × 3^(Trial type: standard, startle, poststartle)^ repeated measures analyses of variance (ANOVA) were carried out. To reduce the risk of a type 1 error because of multiple tests being run on performance, both reaction time and accuracy analyses were carried out with an adjusted significance level of .025. Repeated measure ANOVAs were also used to assess the physiological stress responses, the ER‐SCR following the stimuli, and the HF‐HRV over time. The mean ER‐SCR levels for the stimuli were compared across the three blocks. The test HF‐HRV, measured as the difference in HF‐HRV from the resting baseline, was also compared across the blocks.

Linear multiple regression was used as an analytical tool to examine relations between performance, the physiological stress responses, and neuroticism.

## 4 RESULTS

Since it is possible for a stimuli to sometimes not elicit a startle response for a number of reasons, a simple initial check of the data was conducted to control that physiological responses had been triggered. This analysis showed that the stimuli consistently induced a significant response in the participants: ER‐SCR amplitudes following stimuli had a mean level of 1.80 μS (SD = 1.32) across the blocks, which was significantly higher than spontaneous the SCR fluctuations: *t*(29) = −5.96, *p* < .00). The mean peak heart rate increase was also significant, 15.36 bpm (SD = 4.2): *t*(32) = −2.135, *p *= .041.

### 4.1 CRT performance

**FIGURE 3:**
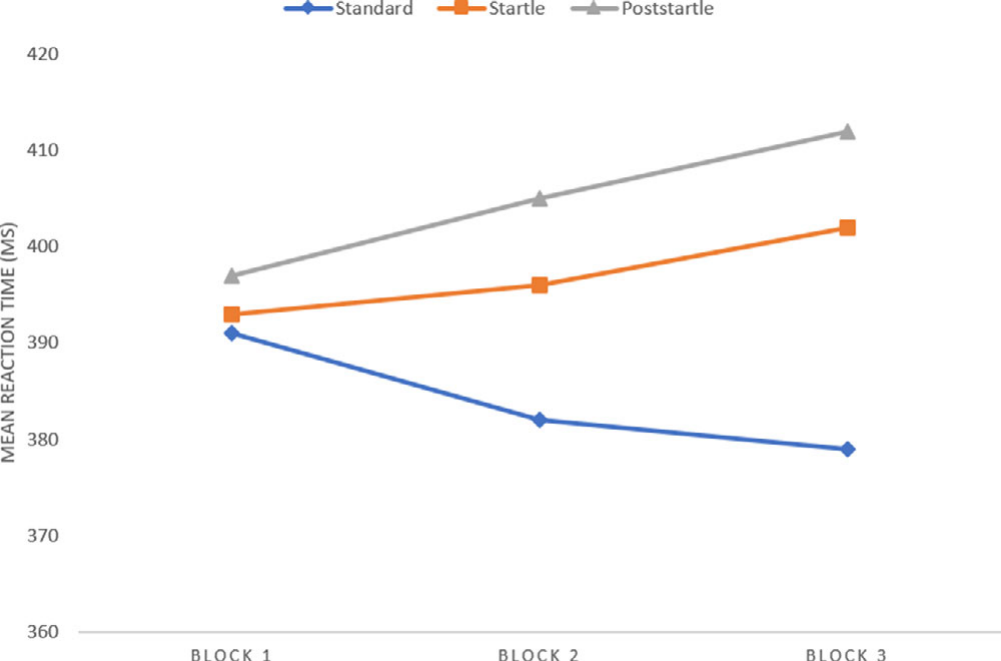
Reaction times in milliseconds for trial type by test block

Reaction times (Figure 3) and accuracy were examined to answer the question whether startling acoustic stimuli affect task performance. Descriptive data are displayed in Table 1.

**TABLE 1:** Means and standard deviations for reaction times and accuracies by block and trial type

Trial type	Measure (*M* ± SD)	Block 1	Block 2	Block 3
Standard	Reaction time (ms)	391 ± 67	382 ± 67	379 ± 65
	Accuracy (%)	91.1 ± 2.6	95.6 ± 4.3	95.7 ± 2.6
Startle	Reaction time (ms)	393 ± 86	396 ± 76	402 ± 96
	Accuracy (%)	88.1 ± 18.1	95.3 ± 9.8	95.3 ± 9.8
Poststartle	Reaction time (ms)	397 ± 117	405 ± 89	412 ± 154
	Accuracy (%)	89.4 ± 7.2	92.5 ± 14.9	97.3 ± 7.7

#### 4.1.1 Reaction time

A 3^(Block: 1, 2, 3)^ × 3^(Trial type: standard, startle, poststartle)^ repeated measures ANOVA for reaction time was conducted. There was a main effect of trial type on reaction time: Wilks's Lambda = .794, *F*(2, 36) = 4.676, *p* = .016, *η*_p_^2 ^= .093, indicating a difference in reaction time between the different trial types. Post hoc Bonferroni corrected *t*‐test of mean reaction times by trial type revealed a significant difference between standard trials and poststartle trials, *t*(37) = −2.667, *p *= .011, revealing that the participants were significantly slower in the poststartle trials than in the standard trials. There were no other significant terms between standard trials and startle trials (*p *= .292) or between startle and poststartle trials (*p* = .124).

There was no significant effect of the block on reaction time: Wilks's Lambda = .992, *F*(2, 36) = .144, *p *> .05, *η*_p_^2 ^= .003, showing no differences in reaction time depending on the block. There was no interaction effect on reaction time from trial type and block either: Wilks's Lambda = .848, *F*(2, 36) = 1.518, *p *> .05, *η*_p_^2 ^= .033. Because the difference in reaction time between the standard and poststartle trials was the only significant effect, further reaction time analysis compared only these variables.

#### 4.1.2 Accuracy

As is visible in Table 1, participants performed accurately overall. A 3^(Block: 1, 2, 3)^ × 3^(Trial type: standard, startle, poststartle)^ repeated measures ANOVA was carried out for accuracy. There was a main effect of the block on accuracy, Wilks's Lambda = .874, *F*(2, 36) = 5.328, *p* = .027, *η*_p_^2 ^= .129, indicating an increase in accuracy over time. Post hoc Bonferroni corrected t‐tests of accuracy by block revealed a significant increase in accuracy between Blocks 1 and 2 as well as Blocks 1 and 3, *t*(37) = −1.607, *p* = .017 and *t*(37) = −2.706, *p* = .01, respectively. There was no significant difference between accuracy in Blocks 2 and 3: *t*(37) = −.212, *p *> .05.

There was no significant effect of trial type on accuracy, Wilks's Lambda = .955, *F*(2, 36) = .847, *p *> .05, *η*_p_^2 ^= .091, suggesting similar accuracy for the different trial types. There was no interaction effect between the trial type and block: Wilks's Lambda = .881, *F*(2, 36) = 2.441, *p* > .05, *η*_p_^2 ^= .024.

### 4.2 Physiological stress over time

A repeated measures ANOVA of ER‐SCR over the blocks indicated that there was a difference in the SCR over time: Wilks's Lambda = .643, *F*(2, 28) = 7.782, *p* = .002, *η*_p_^2 ^= .304. Post hoc tests confirmed that the SCR in Block 1 was significantly higher than in Blocks 2 and 3, *t*(30) = 3.921, *p* < .000 and *t*(30) = −2.936, *p* = .006, respectively. There was no significant difference between SCRs in Blocks 2 and 3, *t*(30) = .738, *p *> .05, indicating a habituation of the stress responses measured with the SCR across the blocks.

A *t*‐test comparing participants’ resting HF‐HRV (*M* = .089, SD = .022) to their HF‐HRV during the experimental condition (*M *= .062, SD = .015) indicated that the HF‐HRV was significantly lower during exposure: *t*(31) = 7.804, *p* < .000, *d* = 1.37. Block‐wise repeated measures ANOVA of the test HF‐HRV did not indicate any difference over time, Wilks's Lambda = .867, *F*(2, 30) = 2.305, *p *> .05, *η*_p_^2 ^= .066, indicating no habituation across the blocks.

### 4.3 Neuroticism, physiological stress measures, and performance

The relation of neuroticism and physiological stress measures to the reaction time effect was modeled using a multiple linear regression. After scoring, participants had a mean neuroticism score of 2.6 on a scale from 1 to 5 (SD = .86) (*α* = .74), very close to the norm value of 2.54 (SD = .80; Donnellan et al., 2006). A model was created using the reaction time difference from the standard to poststartle trials as the dependent variable and neuroticism, mean ER‐SCR, and the test HF‐HRV as independent variables (Table 2).

**TABLE 2:** Results of multiple linear regression analysis for reaction time difference

Variable	B	SE	*β*	*T*	*p*
(Constant)	−25.068	23.240		−1.079	.292
HF‐HRV variation	715.139	365.789	.382	1.955	.063
Neuroticism	6.982	7.122	.198	.980	.338
ER‐SCR	1.068	4.935	.043	.216	.831

*Abbreviations*: ER‐SCR, event‐related skin conductance responses; HF‐HRV, high frequency band heart rate variability.

As shown in Table 2, the standardized coefficient sizes indicate the test HF‐HRV and neuroticism were most informative about reaction time. However, none of the coefficients was statistically significant, but the test HF‐HRV was close. Indeed, although the model was of an acceptable fit (*R* ^2^_adjusted_ = .065), the regression model ANOVA did not indicate the independent variables as significant reliable predictors for the dependent variable, *F*(3, 22) = 1.550, *p *> .05. Constructing models with varying configurations of predictors, such as removing the weaker ER‐SCR variable, did not yield any significantly improved models.

## 5 DISCUSSION

In this study, we examined performance following startle in a visual choice reaction task and the accompanying physiological stress response. We analyzed performance and the physiological stress response over time and across consecutive startles and examined the relation between performance, physiological stress response, and neuroticism. Our results suggest that the unpredictable, distracting startle negatively affected sustained attention performance and that physiological stress followed the stimuli. The distraction effect on attention persisted, in that affected performance was visible across all three test blocks. Although the HF‐HRV measure of physiological stress response remained on similar levels over time, the SCR showed a significant decrease over time. There were no significant relations between effect on performance, biological stress responses, and neuroticism levels.

The analysis of performance data showed that reaction time following startle was slightly negatively affected (≈20 ms), but accuracy was not. These results are in line with previous findings on the cognitive effects following startle (Thackray, 1988; Vlasak, 1969) in that minimal and short‐term effects are present for simple tasks. However, in our study, the effect was not instantaneous in that the trials accompanying the stimuli did not differ from standard trials, but the following trials did. This might be an effect of the time required to process the unpredictable startling acoustic stimuli; although the reflexive part of the startle response is generally initiated within a few milliseconds, the cortical threat assessment has been noted to take upwards of 500 ms sometimes (LeDoux, 2000). It is thus possible that the lack of effect on the startle trial is based on the stage of processing at that time. Granted the short moment between stimuli and trial and that StartReact research has shown the timing of the stimuli might evoke a quicker motor response (Valls‐Solé et al., 1995), this also might be responsible for the lack of visible effect in the startle trials. Consulting the related scientific fields of distraction and interruption, the relatively small effect on task performance may be attributed to the low task demand and complexity. Indeed, the interruption hypothesis of Blumenthal et al. (2015) was suggested from data on a more demanding Attention Network Task, and it is thought that recovery is dependent on the complexity of the situation (Altman & Trafton, 2007). The load theory of attention (Lavie et al., 2004), the findings suggest that the cognitive demands of a task, the cognitive load, is connected to the level of interference from distractors. Lavie et al.’s theory (2004) declares that in a state of low cognitive demand, cognitive control resources monitor priorities to reduce interference from perceived distractors, but in high cognitive demand states, there might be no spare resources to effectively monitor what to ignore, and as such, interference from distractors increases. It is plausible that with the low level of challenge posed by the task in our study, designed to measure reaction time without any added difficulty (Deary et al., 2011), participants had unused cognitive control resources available when they were exposed to the startle. Longer lasting effects observed on tasks of increased complexity, such as by Vlasak (1969) and Woodhead (1959), are supportive of this notion that low task demands result in the effect observed in our study. It would thus be informative for future studies to examine the effects of startle on more cognitively demanding tasks from a perspective of increased workload. Although the mechanics of how startle affects behavior might be partially explained by its similarity to distraction effects, it is pertinent to remember that a startle response is not simply a response to a distraction.

When looking at performance over time, our analysis did not reveal any interaction effect across the blocks. This indicates that the slowing of reaction time following the startles is not something that passes with repeated exposure, nor is there any additive stress from repeated startles that lead to an increase in negative effects over time. Although statistical analysis did not indicate any such effect across the blocks, the descriptive data (as illustrated in Figure 3) indicate a trend toward the latter: incremental slowing of startle and poststartle trials as compared to standard trials. Without speculating too deeply about nonsignificant trends, it is possible that, again, the low complexity of the task hides increased negative effects across exposures that might appear in higher complexity scenarios.

Regarding physiological stress responses, the SCRs following startle were significantly higher than after spontaneous activation, a pattern that persisted for the duration of the task. This is consistent with general patterns of physiological activity following startling or affective events (Bradley et al., 1993) and with studies on cognitive performance and startle (Thackray, 1988). There was a decrease in this response over time, indicative of a level of habituation to the stimuli. However, this sign of habituation was not reflected in the measured reaction time. The HF‐HRV was significantly lower than the baseline for the duration of the test, and it remained stable across the blocks. This could indicate that the observed difference in HF‐HRV in this case is more a measure of the cognitive effort of performing the task rather than the response of the stimuli. When combined with the reaction time data, our results do not indicate a correlation between the physiological response to the startling stimuli and its effect on task performance. This stands in contrast to earlier results (Thackray, 1988) where SCR and heart rate correlated to the level of performance effect. As the relation between performance effect and HF‐HRV was almost significant, it is possible that some confounding factors were hiding an effect that might have appeared in a slightly different situation. It might be that because of the low cognitive load, as discussed previously, every participant had the spare cognitive resources to negate all but the smallest impairment, regardless of their stress level. Using more complex tasks with higher demands could elucidate whether there are relations between performance effects and the physiological stress response such as those discussed in Thackray (1988). On a broader level, the homogenous nature of the participant sample (all participants were recruited from a university environment and of a relatively young age) might well be involved in these outcome results. Indeed, higher education is positively related to cognitive performance (Guerra‐Carrillo et al., 2017), and reaction time as an aspect of attention is well known to follow a negative trend with age (Deary & Der, 2005). It is then possible that different patterns such as a grander effect might be visible in samples of older age or when recruited elsewhere.

One of the key questions in this study was to investigate the role of neuroticism and its relation to stress under startle. Our results suggest that the degree of neuroticism does not appear to be related to the performance following startling stimuli. Based on the nature of this study, too strong conclusions about the irrelevance of this manner of personality trait should not be drawn. Indeed, regarding the connection between higher neuroticism and increased physiological stress, research has been conducted mostly on stimuli of some level of emotional valence (Hur et al., 2016; Wilson et al., 2000) or in more ecologically valid situations. The lack of relation between performance and neuroticism could then again be dependent on the simplicity of the task, the laboratory setting, and the emotional valence of the currently used stimulus. Managing the nature of the induced startle to involve a negative affect might not only better inform the relationship between cognitive performance effects and neuroticism but also lay better ground for analyzing performance effects in general. It has been known for some time that responses to startling stimuli increase within threatening situations (Grillon et al., 1991), and the inclusion of this fear might be enough to bring out more noticeable effects on even base cognition. In addition, using instruments to measure more specific aspects of neuroticism might also prove informative. Anxiety, a subfactor of neuroticism, might be a relevant candidate as well. High anxiety encompasses a sensitivity to threat (see Cisler & Koster, 2010, for a review) and is related to the startle response magnitude (Poli & Angrilli, 2015). Our sample was also relatively homogenous when considering levels of neuroticism, and research on the effects of neuroticism often targets populations high in the trait (Di Simplicio et al., 2012; Larsen & Ketelaar, 1991). As such, specifically recruiting highly neurotic participants and/or establishing a comparison to a control group might provide results unavailable in this initial examination. Additionally, it cannot be ruled out that the lack of visible relations between the stress response and neuroticism as well as performance could in some part be due to the sample size of the current study.

Investigating startle in relation to cognitive performance is a complicated issue because of the high number of measurement points that is usually desired for stable statistical analysis. Repeated exposures to stimuli can decrease its surprising nature (Teigen & Keren, 2003), and repeated exposures to startling stimuli normally lead to a habituation of the reflexes (Geyer & Braff, 1982). This creates a dilemma when designing a study on repeated measurements: An increase in power might be accompanied with an increased risk for habituation effects. We opted for a repeated exposure design with the benefit of decreasing variances between individuals and aimed to minimize habituation by randomly distributing the startle trials across the test. Varying lengths of recovery time were used between the stimuli, which might have affected the level of startle response to some stimuli. However, the results indicated stable performance effects even when considering this, which could be seen as a strength in our results.

In conclusion, based on the results of this study, a startling acoustic stimulus degraded performance in a sustained attention task. This effect appeared to be independent from the level of physiological stress as measured by the SCR, HF‐HRV, and neuroticism level. The low task demands may be a contributing factor to the obtained results. Thus, although these results aid in quantifying the effects of startling acoustic stimuli on basic cognition, further studies are needed to examine effects on tasks with a higher level of cognitive workload, where cognitive effects might appear more pronounced and stress responses might be reflected in the performance.

## CONFLICT OF INTEREST

The authors have no conflict of interest to declare.

### PEER REVIEW

The peer review history for this article is available at https://publons.com/publon/10.1002/brb3.2554

## Data Availability

Due to the nature of this research, participants of this study did not agree for their data to be shared publicly, so supporting data is not available.
